# Carbohydrates and carbohydrate degradation gene abundance and transcription in Atlantic waters of the Arctic

**DOI:** 10.1038/s43705-023-00324-7

**Published:** 2023-12-09

**Authors:** Taylor Priest, Silvia Vidal-Melgosa, Jan-Hendrik Hehemann, Rudolf Amann, Bernhard M. Fuchs

**Affiliations:** 1 Institute of Microbiology, ETH Zurich, Zurich, Switzerland; 2 Max Planck Institute for Marine Microbiology, Bremen, Germany; 3 University of Bremen, MARUM, Bremen, Germany

**Keywords:** Microbial ecology, Microbial ecology, Metagenomics, DNA sequencing, Transcriptomics

## Abstract

Carbohydrates are chemically and structurally diverse, represent a substantial fraction of marine organic matter and are key substrates for heterotrophic microbes. Studies on carbohydrate utilisation by marine microbes have been centred on phytoplankton blooms in temperate regions, while far less is known from high-latitude waters and during later seasonal stages. Here, we combine glycan microarrays and analytical chromatography with metagenomics and metatranscriptomics to show the spatial heterogeneity in glycan distribution and potential carbohydrate utilisation by microbes in Atlantic waters of the Arctic. The composition and abundance of monomers and glycan structures in POM varied with location and depth. Complex fucose-containing sulfated polysaccharides, known to accumulate in the ocean, were consistently detected, while the more labile β-1,3-glucan exhibited a patchy distribution. Through ‘omics analysis, we identify variations in the abundance and transcription of carbohydrate degradation-related genes across samples at the community and population level. The populations contributing the most to transcription were taxonomically related to those known as primary responders and key carbohydrate degraders in temperate ecosystems, such as NS4 Marine Group and *Formosa*. The unique transcription profiles for these populations suggest distinct substrate utilisation potentials, with predicted glycan targets corresponding to those structurally identified in POM from the same sampling sites. By combining cutting-edge technologies and protocols, we provide insights into the carbohydrate component of the carbon cycle in the Arctic during late summer and present a high-quality dataset that will be of great value for future comparative analyses.

## Introduction

Marine carbohydrates are chemically and structurally diverse, and represent a substantial fraction of characterised organic matter [1]. The diversity of glycans emerges from the alternative linkage types, alpha and beta, of carbon atoms between more than ten available monomers along with substitutions by a range of other chemical groups [2]. Micro- and macro-algae are the primary synthesisers of glycans in the ocean, wherein they serve structural, storage and protective functions. Glycans can constitute between 13 – 90% of algal carbon [3]. Marine glycans range from low-molecular weight (LMW) oligosaccharides to complex high-molecular weight (HMW) polysaccharides, with varying composition across taxa, life-cycle stage and environmental conditions [4, 5]. Through exudation, cell death and lysis, various glycans are released and integrated into the particulate and dissolved organic matter pools (POM and DOM) [6–8], which are separated based on the size of particles. Once released or outside the cell, glycans can become substrates for heterotrophic microbes.

Carbohydrate utilisation is common in bacteria and archaea, but the mechanisms employed and the degradative capabilities vary [9–11]. Many species take up mono-, di- and trisaccharides into the cell through porins or transporters, while longer oligosaccharides require specialised systems, such as TonB-dependent transporters (TBDTs) or other outer membrane proteins. Usually these have a high specificity to discrete glycan structures [12]. For polysaccharides, microbes must first depolymerise the structure extracellularly with excreted or outer membrane-bound glycoside hydrolases (GHs) or polysaccharide lyases (PLs), followed by uptake of the oligosaccharides and subsequent cleavage in the periplasm [12, 13]. These enzyme classes, together with carbohydrate-binding modules (CBMs) and carbohydrate esterases (CEs), are collectively referred to as carbohydrate-active enzymes (CAZymes). CAZymes are classified into families based on protein sequence similarity, with each family also containing at least one biochemically characterised protein [14, 15]. Many families are monospecific to certain glycosidic linkage types within polysaccharides while others are divided into sub-families based on specificity of target linkages [16]. Unlike those from land plants, algal glycans are often decorated with sulphate esters, which require sulfatase enzymes for complete degradation. The CAZyme, sulfatase and transporter gene profiles thus acts as the blueprint for the glycan degradation potential of microbes [10, 17].

Carbohydrate utilisation by microbial populations exhibit spatial and temporal variations. The rate of hydrolysis and the substrate spectrum of extracellular CAZymes decreases and narrows with depth [18] and with distance to the coast [19]. In addition, a broader spectrum of CAZyme activities is measureable in temperate compared to high-latitude waters, indicating latitudinal gradients [20]. Temporal shifts in CAZyme, sulfatase and transporter gene profiles are also evident following spring phytoplankton blooms [21]. These patterns are congruent with dynamic changes in microbial community composition. In particular, community-level patterns are shaped by the presence and composition of specialised carbohydrate degraders, such as *Bacteroidetes. Bacteroidetes* typically harbour large CAZyme repertoires [10, 17] and exhibit successional dynamics following spring phytoplankton blooms [21]. These dynamics indicate glycan-based niche partitioning [22, 23]. Detailed assessments on microbial carbohydrate utilisation have been focused on spring phytoplankton blooms in temperate ecosystems, while far less is known about later seasonal stages and higher latitude waters.

In this study, we combine analytical techniques with meta’omics approaches to explore the distribution of glycans and their potential utilisation by microbes in Atlantic waters of the Arctic during late summer. The carbohydrate composition of particulate organic matter (POM) in the upper euphotic zone was characterised using glycan microarrays and high-performance anion-exchange chromatography. Concurrently, we applied PacBio HiFi read metagenomics and Illumina short-read metatranscriptomics to assess the abundance and transcription of carbohydrate degradation-related genes in microbial communities. By combining cutting-edge techniques and protocols, we aim to provide insights into the carbohydrate component of the carbon cycle in high-latitude waters during late summer.

## Methods

### Sample collection

**Fig. 1:**
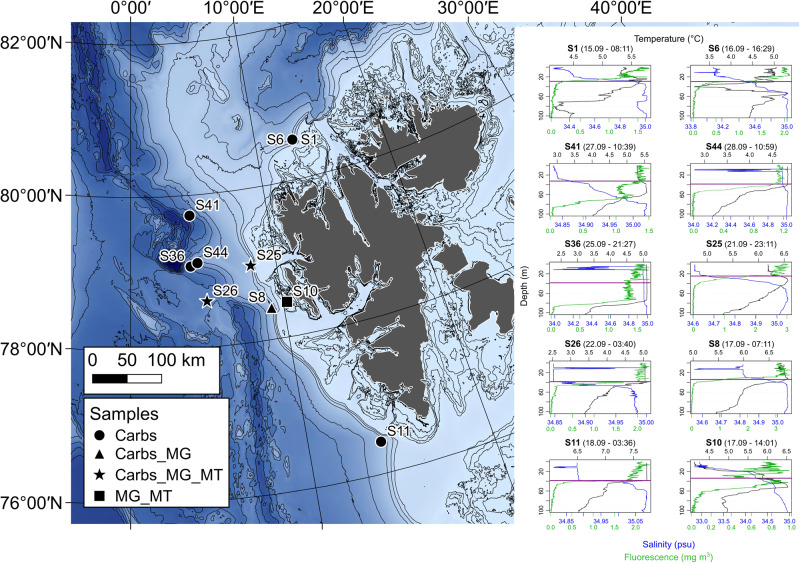
Bathymetric map with sampling locations, types of samples collected and vertical profiles from CTD casts. Map legend: Carbs = samples collected for carbohydrate analysis on particulate organic matter fraction, MG = sampled for metagenomics, MT = sampled for metatranscriptomics. Vertical profiles of temperature, salinity and fluorescence were derived from sensor measurements during CTD casts. The purple horizontal lines on each profile represent the bottom mixed layer (BML) sampling depth.

Seawater samples were collected from ten stations located in the eastern Fram Strait and around the Svalbard archipelago in September 2020 during the MSM95 Maria S. Merian research cruise. A map of the sampling locations (Fig. 1) was generated using publically available bathymetric data from the International Bathymetric Chart of the Arctic Ocean (IBCAO) [24] and the QGIS v3.14.16-Pi [25] software. Seawater was collected using a CTD-rosette sampler from surface water (SRF), typically 2 m depth, and the bottom of the surface mixed layer (BML). The BML depth was defined by the beginning of the thermo- and halocline and drop in surface fluorescence values (Fig. 1). One location was sampled twice over a two day period, with additional samples collected at 100 and 200 m depth, these are labelled as S1 and S6 (Supplementary Table S1). Of the water collected, 4 L was filtered sequentially through a 3 and 0.2 µm pore-size polycarbonate membrane filter (142 mm diameter) and immediately stored at −80 °C for ‘omics analysis. A second 4 L of seawater was filtered through a 0.7 µm pre-combusted Whatman Grade GF/F filter (47 mm diameter) and immediately stored at -80 °C for carbohydrate analysis.

### Monosaccharide and polysaccharide analysis

The GF/F filters from all samples (ten stations and two depths) were cut into ten equally-sized circular pieces, with a diameter of 10 mm. The monosaccharide composition of and polysaccharide structures present on the filter pieces was analysed as described previously [26] and detailed in Supplementary Methods. First, two of the filter pieces were hydrolysed using acid (1 M HCI for 24 h at 100 °C) and the resulting monosaccharides were analysed using High-Performance Anion Exchange Chromatography with Pulsed Amperometric Detection along with monosaccharide standards. Acidic monosaccharides could not be detected due to a problem with the detector. The remaining filter pieces were subject to polysaccharide extraction using a sequential solvent protocol, with 1) MilliQ water, 2) 0.2 M EDTA (pH 7.5) and 3) 4 M NaOH with 0.1% w/v NaBH_4_. The identification and semi-quantitative analysis of polysaccharide compounds was performed using a microarray and antibody-based approach. Polysaccharide extracts were printed in quadruplicates onto nitrocellulose membranes (0.45 µm pore-size) using a microarray robot (Sprint, Arrayjet, Roslin, UK). The printed arrays were blocked for 1 h in 1 x PBS with 5% (w/v) non-fat milk powder (MPBS), followed by incubation for 2 h with polysaccharide-specific monoclonal antibodies (Supplementary Table S2). After incubation, the arrays were washed in PBS and incubated for 2 h in anti-rat, anti-mouse or anti-His tag secondary antibodies conjugated to alkaline phosphatase. Arrays were thoroughly washed in PBS and deionized water before being developed in a solution containing 5-bromo-4-chloro-3-indolylphosphate and nitro blue tetrazolium in alkaline phosphatase buffer (100 mM NaCl, 5 mM MgCl_2_, 100 mM Tris-HCl, pH 9.5). Developed arrays were scanned and the binding of each probe against each spotted sample was quantified using Array-Pro Analyser 6.3 (Media Cybernetics). Signal intensities for each extract against each antibody were quantified. The highest signal value in the data set (which corresponded to a standard control) was set to 100 and all other values were normalised accordingly. Only antibodies with a signal >=5 in at least one sample were retained. Mean signal intensities (from the four replicates) were then determined for each antibody in each sample.

### Metagenome and metatranscriptome sequencing

Filtered seawater samples of the 0.2 – 3 µm fraction from SRF and BML depths of four different stations (S8, S10, S25, S26) were subject to a dual nucleic acid isolation protocol using the DNA/RNA Mini Prep Plus kit from Zymo Research (Irvine, CA, USA), according to the manufacturer’s instructions. The quality of extracted DNA was assessed using capillary electrophoresis with a FEMTOpulse (Agilent), whilst RNA quality was assessed using a PicoChip on a Bioanalyser (Agilent, CA, USA). Ultra-low DNA libraries were prepared from the eight samples without further fragmentation by the protocol “Procedure & Checklist - Preparing HiFi SMRTbell® Libraries from Ultra-Low DNA Input” of PacBio (CA, USA). Libraries were sequenced on 4 x 8 M SMRT cells on a Sequel II platform for 30 h with sequencing chemistry 2.0 and binding kit 2.0 (two samples multiplexed per SMRT cell). Four of the samples were additionally selected for metatranscriptome sequencing (S10_SRF, S25_SRF, S25_BML, S26_BML). Illumina-compatible libraries were produced from extract RNA using the Universal Prokaryotic RNA-Seq library preparation kit, incl. Prokaryotic AnyDeplete® (Tecan Genomics, CA, USA). Libraries were sequenced on a HiSeq 3000 platform with 2 ×150 bp paired-end read mode.

### HiFi read taxonomic classification

A custom pipeline was employed to taxonomically classify metagenomic HiFi reads against a GTDB-based protein database. A Diamond blast (v0.9.14) [27] database was generated from the gene amino acid sequences of all GTDB species-representatives (release 207) after clustering at 99% sequence identity, to remove redundancy. NCBI-style taxdump files (nodes.dmp, names.dmp and accession2taxid) were then generated using scripts from https://github.com/nick-youngblut/.

Open reading frames were predicted on raw HiFi reads using FragGeneScan v1.31. Gene sequences were aligned to the generated GTDB protein database using Diamond blastp (parameters: --id 50 --top 5 --fast). A secondary filtering step was applied to the output including identity threshold >65%, e-value < 1E-10 and query-cover >50%. Using the remaining hits, a single taxonomic classification for each gene was determined using a last common ancestor approach, *lca* command from TaxonKit [28]. To further increase the number of genes taxonomically classified, the last common ancestor algorithm was then applied to all genes within a single HiFi read, resulting in a single taxonomic classification for each HiFi read, and its containing genes.

### HiFi read functional annotation

For functional characterisation, the predicted gene sequences (see above) from HiFi reads were subject to a custom annotation pipeline, modified from Priest et al. [29]. In brief, genes were annotated against the Pfam database (release 35.0) using HMMsearch v3.3.2 (parameters: cut_ga), UniProtKB database (05.2022) using Diamond blastp v2 (parameters: -k 1 --evalue 1e-10 --query-cover 50 --id 40 --sensitive) and the KEGG database (07.2022) using kofam_scan (https://github.com/takaram/kofam_scan; parameters: -E 0.0001). Additional annotations were obtained by searching more specialised databases using HMMScan (Transporter Classification database; obtained 11.2021, TonB HMM profiles from TIGRFAM, and dbCAN; v10) and Diamond blastp (CAZyDB; release 09242021, SulfAtlas; v1.3, MEROPS; v12.1) using the same settings as described above, except for HMMScan against the dbCAN database (parameters: -E 1E-15).

### Single-copy ribosomal protein (SC-RBP) gene analysis

From the gene annotations, 16 single-copy ribosomal protein (SC-RBP) genes [30] were identified and extracted from each metagenome. The average number of SC-RBP was used as a proxy for the number of genomes recovered in each metagenome. A subset of four SC-RBP genes (RBP L3, L4, L6 and S8) were clustered at previously defined gene-specific ANI thresholds [31] and the average number of clusters across the four was used as a proxy for the number of species captured. The composition of metagenomes and metatranscriptomes was compared based on the species clusters from the RBP L6 gene, selected due to its high recoverability and species delineation accuracy [31]. The taxonomy of each cluster was determined based on a majority vote between the taxonomy of the contained genes, derived from original HiFi read classifications.

### Assembly, binning and metagenome-assembled genome recovery

The assembly of Hifi reads was performed using MetaFlye v2.8 [32] (parameters: --meta –pacbio-hifi –hifi-error 0.01 –keep-haplotypes). Coverage information was obtain through mapping HiFi reads to assembled contigs using Minimap2 v2.1 (parameters: -x map-hifi –MD). Contigs were binned using Metabat2 [33]. The resulting bins were subject to manual refinement using the Anvi’o v7 [34] interactive interface to generate metagenome-assembled genomes (MAGs). MAGs were dereplicated at a 99% ANI threshold using dRep v3.2.2 (parameters: --comp 50 --con 5 --sa 0.99 --nc 0.6). The completeness and contamination of representative MAGs was estimated using CheckM v1.1.2 [35]. A two-pronged approached was used for taxonomic classification of MAGs, the classify_wf pipeline of GTDB-tk v1.0.2 [36] (Release 207) and the extraction of 16 S rRNA gene sequences using Barrnap v0.9 [37] and classification against the SILVA_SSU_Ref138.1_NR99 database, following the same process described in ‘*Phylogenetic characterisation of communities’*. Of the species-representative MAGs, 84% contained a complete 16 S rRNA gene and thus received dual taxonomies.

### MAG relative abundance estimation

The relative abundance of representative MAGs was determined using a similar approach to Orellana et al. [38]. In brief, reads were competitively recruited from each metagenome to the MAG representatives. Mapped reads were converted into depth values using Genomecov (-bga option) from the Bedtools package [39] and the 80% central truncated average of the sequencing depth (TAD) was determined using the ‘BedGraph.tad.rb’ script (option range 80) from the enveomics collection [40]. The relative abundance was then determined as the quotient between the TAD value and the number of microbial genomes captured in each metagenome, determined from SC-RBP genes.

### MAG functional characterisation

The functional characterisation of MAGs was performed following the same procedure described for the HiFi reads above except for an additional process of polysaccharide utilisation loci (PULs) detection. PULs were defined as genetic loci containing a SusC/SusD gene pair with two or more degradative CAZymes or the presence of at least three degradative CAZymes in close proximity (maximum six genes apart). PULs were manually inspected and visualised at BioRender.com.

### Transcription level of genes at the community- and MAG-level

Adaptors and low quality reads were removed from the metatranscriptomes using BBDuk of the BBtools programme v38.73 [41] (parameters: ktrim=r, k = 29, mink=12, hdist=1, tbo=t, tpe=t, qtrim=rl, trimq=20, minlength=100). Although an rRNA depletion step was performed prior to sequencing, it is expected that 5 – 15% of reads would still be related to rRNA. As such, SortMeRNA v2.0 [42] was used to filter out rRNA sequences from the dataset, with the SILVA SSU Ref 138 NR99 database as a reference. The transcription level of genes was determined by read recruitment of transcripts to the predicted gene sequences from the HiFi reads using BBmap (v35; parameters: minid=98 idfilter=98). Mapped read values were converted to transcripts per million (TPM), according to Wagner et al. [43]. For MAGs, transcripts were competitively recruited to all MAG genes, with same procedure as above, and the values were converted to TPM using the total number of transcripts recruited to the whole metagenome-predicted genes as the total transcript values. In order to compare the transcription level of MAGs across samples, we determined the average TPM value of the 16 SC-RBPs for each MAG in each sample, and took the quotient of this and the average TPM value of the 16 SC-RBPs in the whole sample – providing proportional transcription of all genomes recovered. To place MAG CAZyme gene family transcription into the context of the whole community, we performed an additional read recruitment step. First, we concatenated genes from all MAGs into files based on CAZyme gene family or sub-family annotations. Then, we identified all transcripts that mapped to the metagenomic read-predicted genes for each of these families and subsequently recruited them to the concatenated MAG gene files, using the same parameters assigned above. Based on the number of transcripts mapped, the relative proportion of CAZyme gene family transcription was determined for each MAG.

## Results & discussion

Seawater samples were collected from surface waters (SRF) and the bottom of the surface mixed layer (BML) in the Eastern Fram Strait region to investigate the distribution of carbohydrates and their utilisation by microbial communities. The ten sites were grouped into three categories based on the underlying seafloor topography (above-slope, above-shelf and open-ocean), which also corresponded to differences in hydrographic conditions. The main water mass in this region is of North Atlantic origin. The West Spitsbergen Current (WSC) transports Atlantic water northward into the Arctic Ocean, with the main branch flowing above the continental slope. At the shelf break, a temperature-salinity front occurs, whilst above the West Spitsbergen shelf, Atlantic water (AW) converges and mixes with Arctic water and freshwater from land, resulting in intra-annual variability in hydrographic properties [44]. Based on temperature and salinity values, the main water masses in this region can be distinguished, with AW characterised by >34.9 psu and >4.1 °C [45]. The temperature of SRF and BML depths during sampling in this study were indicative of AW, ranging from 4.1 – 7.7 °C. However, the salinity values in SRF waters of above-shelf (S10) and three above-slope stations (S1, S6 and S8) were below the AW-defining thresholds (Fig. 1 and Supplementary Table S1). These observations suggest an influence of either polar-derived water or freshwater from Spitsbergen at these stations.

### Carbohydrate analysis of POM samples

**Fig. 2:**
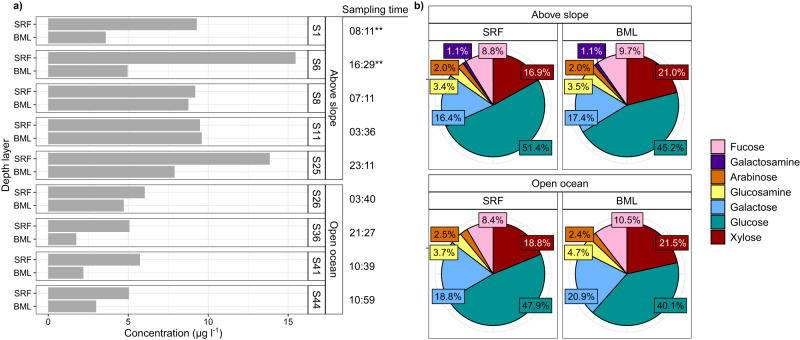
Total concentration and relative abundance of monosaccharides in carbohydrates from POM fraction. **a)** Total monosaccharide concentrations as ug per L of seawater at each station and depth**, b)** Relative abundance of monosaccharides grouped by station location in relation to continental slope. Data show neutral and amino monosaccharides. SRF = Surface water sample, BML = sample from bottom of the surface mixed layer.

The monosaccharide and glycan composition of carbohydrates in POM ( > 0.7 µm) was analysed in SRF and BML depths at nine stations. The monosaccharide composition of carbohydrates in POM varied with depth and location (Fig. 2). Total neutral and amino monosaccharide concentrations ranged from 1.4 – 13.8 µg per L of seawater (hereon µg l^−1^). Higher values were typically observed in SRF, average of 8.8 µg l^−1^, compared to BML depths, average of 5.2 µg l^−1^ (Supplementary Table S3). However, the magnitude of change between the two depths was station-dependent, from a negligible difference at station S8 to a threefold decrease from SRF to BML depths at station S6 (Fig. 2a). The decrease in monosaccharide concentrations with depth is in agreement with previous observations from the Pacific Ocean [46]. In addition, above-slope stations contained higher monosaccharide concentrations than open-ocean stations (Fig. 2 and Supplementary Figure S1). This spatial heterogeneity resembles that of chlorophyll *a* and dissolved organic compounds during early summer in this region, which reach highest concentrations in SRF depths above the continental slope ( ~ 8 °E) [47]. These patterns likely reflect hydrographic processes, such as the frontal zone situated above the shelf break.

The most abundant monosaccharide detected in all samples was glucose. Glucose represented a larger relative proportion of POM carbohydrates in SRF ( ~ 49%) than BML ( ~ 43%) depths and in above-slope ( ~ 47%) compared to open-ocean ( ~ 44%) samples (Fig. 2b). These values are within the range of those previously reported from oceanic surface waters (31 – 55%) [48–50] but lower than during phytoplankton blooms, wherein glucose can constitute >70% of POM carbohydrates [51]. Furthermore, in contrast to findings from the high North Pacific [48], the relative proportion of glucose decreased with depth, concurrent with an increase in all other monosaccharides. In particular, xylose increased 5% in relative proportion to the other monomers from SRF to BML depths (Fig. 2b). The relative decrease in glucose with depth could result from a number of factors, including recent production and retention of glucose-containing glycans in surface waters as well as the selective utilisation of these compounds.

**Fig. 3:**
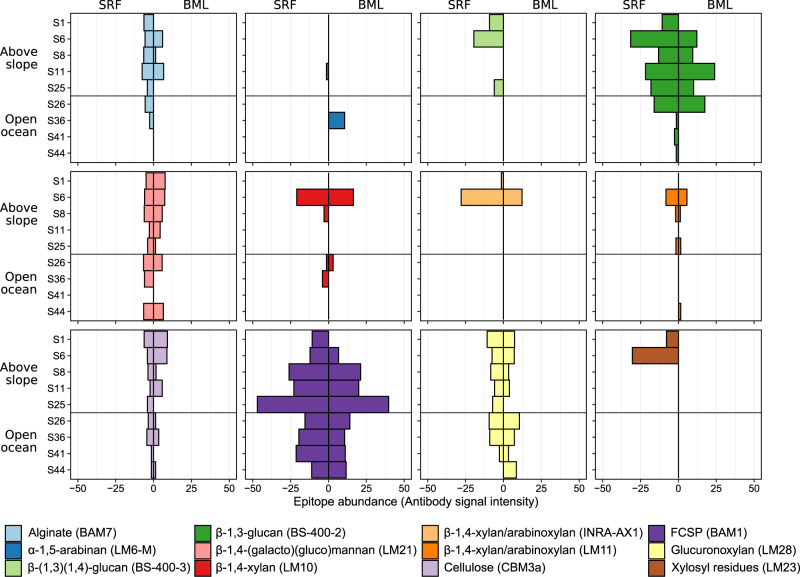
Diverse glycan structures occur in Atlantic waters of the Arctic. Plots show the relative abundance (antibody signal intensity) of polysaccharide epitopes detected in POM samples. Signal intensities for each extract against each antibody were quantified. The highest signal value in the data set (which corresponded to a standard control) was set to 100 and all other values were normalised accordingly. Only antibodies with a signal >=5 in at least one sample were retained. Mean signal intensities (from the four replicates) were then determined for each antibody in each sample. The epitope abundances shown here represent the summed values from the mean signal of H_2_O, EDTA and NaOH extractions. Each antibody has a different avidity, thus the absolute numbers should not be compared between probes to infer different concentrations, but signal from a single antibody determines the relative abundance of the epitope within the sample set. Specific polysaccharide structures that the molecular probes (depicted in parentheses) bind to are shown in the legend. SRF = Surface water sample, BML = bottom of the surface mixed layer. FCSP = Fucose-containing sulphated polysaccharide. Some epitopes that were only detected at low intensity in <3 samples were not included in this plot.

Combining carbohydrate microarrays with monoclonal antibodies and carbohydrate binding modules resulted in the detection of 16 distinct glycan epitopes in POM (Supplementary Figure S2 and Supplementary Table S4). This structure-based detection provides semi-quantitative presence and abundance of distinct epitopes, where changes in antibody binding signal correlates to epitope concentration in the sample [26, 52]. Variations in the abundance of glycan epitopes exposed location- and sample-specific patterns. The glycan epitopes observed most frequently included glucuronoxylan in 91% and fucose-containing sulfated polysaccharide (FCSP) in 82% of samples (Fig. 3). FCSPs are unique to brown algae, wherein they serve important structural roles [53] and formulate part of the secreted carbon pool [54], while glucuronoxylans are common features of land plants [55]. Recently, we discovered both of these complex polysaccharides in microalgal blooms [26] as well as the secretion of FCSPs by diatoms in culture [56]. FCSPs synthesised by diatoms accumulate in POM over a period of weeks during a spring phytoplankton bloom, indicating stability against bacterial degradation [26], and can contribute to long-term carbon sequestration in sediments [57]. This observed stability contrasts β-1,3-glucans, such as laminarin, that are also synthesised by brown algae and diatoms. Laminarin is structurally simpler than FCSPs, with fewer unique linkages and without sulphate esters. Concurrently, the presence and activity of laminarases is observed more frequently in marine microbes compared to those targeting FCSPs, suggesting higher consumption and turnover [10, 26, 58]. In our samples, laminarin was absent at some open-ocean stations, but present in all above-slope sites (Fig. 3). In addition to a potentially more rapid utilisation by microbes, the heterogeneous distribution of laminarin may also result from variations in the distribution of phytoplankton, reflecting previous observations from this region [59].

Sampling conducted at the same location over a two day period (stations S1 and S6) and into deeper waters (down to 200 m) showed additional differences over time and with depth in the abundance and diversity of POM carbohydrates (Supplementary Figure S4). S1 samples were retrieved at 08:00 whilst S6 samples were collected one day later at 16:30. The absolute concentration of monosaccharides in POM carbohydrates was 1.1 – 3.6x higher in S6 compared to S1 samples. In addition, SRF depths in S6 samples contained more α-1,5-arabinan and β-1,3-glucan as well as β-1,4-xylan epitopes and xylosyl residues. The only epitopes more abundant in S1 samples were alginate and glucuronoxylan. It is important to note that the alginate-targeting antibody, BAM7, has cross reactivity with FCSP [60]. The variations in glucose and β-1,3-glucan between the two sampling time points may reflect diurnal fluctuations in laminarin production by diatoms [61], with production during the day and a partial consumption at night. Such diel periodicity has also been shown at the particulate organic carbon level [62], with accumulations during the day to a maximum concentration at dusk [63]. Furthermore, the higher abundance of xylosyl residues and β-1,4-xylan epitopes at S6 could suggest a change in the primary producers between the time points, resulting from shifts in water mass dynamics. It is important to note that these are observations derived from sampling only two time points, and we recognise that the limited replication possibility during sampling and capacity to further sample over temporal scales limits the ecological conclusions that can be drawn.

### Sampled microbial communities were indicative of summer in high-latitude Atlantic waters

To assess microbial carbohydrate utilisation potential, eight PacBio HiFi read metagenomes were generated from SRF and BML depths of two above-slope (S8 and S25), one open-ocean (S26) and one above-shelf (S10) station. From four of the samples, metatranscriptomes were also generated (Supplementary Table S5 and S6). The above-shelf station S10 was chosen for sequencing even though POM carbohydrate samples were unavailable, due to the lower salinity values observed (psu < 33) that indicates influence from polar marine/freshwater and thus provides a contrast for comparison. Despite the limited scope of our sampling scheme and inability to obtain replicates, the employment of two complimentary, cutting-edge sequencing technologies and protocols resulted in the generation of a high-quality ‘omics dataset that can be used to gain valuable insights into microbial carbohydrate utilisation potential.

To place the sampled communities into context, we performed a taxonomy-independent comparison to previously published metagenomes from the Fram Strait [29] and Arctic Ocean [64]. Based on sequence composition dissimilarity, our metagenomes were most closely related to those previously generated from WSC, high North Atlantic and Barents Sea samples in June and July and most dissimilar to those from the polar water mass of the western Fram Strait (Supplementary Figure S5). This indicates that the communities captured in our metagenomes are representative of Atlantic waters of the Arctic during summer.

As our focus was on microbial communities, we removed metagenomic reads not classified as Bacteria or Archaea. Despite the size fractionation employed during sampling (0.2 – 3 µm), 22– 49% of the metagenomics reads were classified as Eukarya. Although our analysis was concentrated on the prokaryotic fraction, we also extracted and analysed 18 S rRNA genes to provide insights into the eukaryotic taxa present in samples (Supplementary Figure S7). Using the average sequencing depth of single-copy ribosomal protein (SC-RBP) genes, we determined the number of microbial genomes sequenced to range from 761 in S8_SRF to 1467 in S10_BML (Supplementary Figure S6b). The number of genomes detected is used to normalise the abundance of functional genes.

### Composition of metagenome and metatranscriptome microbial communities

**Fig. 4:**
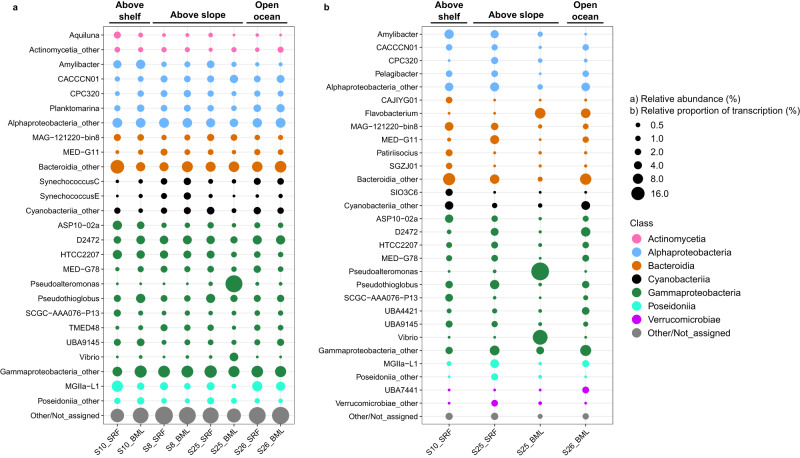
Composition of microbial communities in metagenomes and metatranscriptomes were sample-specific. **a** Relative abundance is described as the relative proportion to total ribosomal protein L6 sequences identified in the sample. **b** Relative proportion of transcription is determined as the relative proportion to total sample ribosomal protein L6 TPM values. Taxonomy of ribosomal protein L6 sequences was derived from taxonomic classification of the original HiFi read, which was classified using a GTDB-based database.

The composition and structure of microbial communities varied across samples. The most prominent families observed in the metagenomes were *Flavobacteriaceae* (4 – 14%), *Rhodobacteraceae* (3 – 13%), D2472 (SAR86; 5 – 12%) and *Poseidoniaceae* (2 – 12%) except for in S25_BML, which was enriched in *Alteromonadaceae* (25%) (Supplementary Figure S8). These bacterial families were also substantial contributors to community transcription. However, the relative proportions of abundance and transcription were not consistent, e.g. *Flavobacteriaceae* represented a two- to threefold higher proportion of transcription. Twenty genera were identified as reaching >2.5% relative abundance and together, constituted ~42% of the microbial communities (Fig. 4). These 20 genera included *Pseudoalteromonas* (0 – 29%), MGIIa-L1 ( < 0.1 - 10%), *Amylibacter* (<0.1 – 6%), D2472 (SAR86; 3 – 6%), ASP10-02a (<0.1 – 6%) and HTCC2207 (SAR92; 1 – 6%). However, only some of these genera were substantial contributors to community transcription. The maximum relative proportion of transcription was observed in *Pseudoalteromonas* (45%), *Vibrio* (29%), *Flavobacterium* (13%), *Amylibacter* (9%), SAR86 (9%) and *Pseudothioglobus* (9%). Discrepancies between metagenomes and metatranscriptomes highlights the disconnection between gene abundance and transcription, as could be expected. The magnitude of discrepancy varied across taxa, with the MGIIa-L1 (Marine Group II Archaea) representing a larger proportion of gene abundance than transcription, while genera within *Bacteroidia*, such as *Flavobacterium* and MAG121220-bin8 (NS4), showed the opposite trend. Such patterns are not uncommon in marine microbial communities [65] and can be influenced by lifestyle, fitness and differences in metabolism.

Sample S25_BML was evidently an outlier in the dataset, harbouring a community dominated by *Pseudoaltermonas* and *Vibrio*. These genera have previously been observed under nutrient-rich conditions and are known to be associated with eukaryotic hosts and phytoplankton blooms [66, 67]. Considering the high proportion of eukaryotic reads captured in some of the metagenomes, the observed pattern in S25_BML may represent signals of processes occurring in the larger size fraction. In support of this, the eukaryotic community of this sample (Supplementary Figure S7) contained a large proportion of 18 S rRNA genes affiliated with copepods, whose microbiomes are often enriched with *Pseudoalteromonas* and *Vibrio* [68, 69]. Therefore, we assume that sample S25_BML was influenced by the presence of copepod faecal pellets on the filter, and thus it represents an outlier that ship-based, point-sampling schemes, such as that employed here, are susceptible to.

In general, the microbial community compositions observed here more closely resemble those previously described from summer [70, 71] than from the sampling time period (late September) in this region [72]. The difference likely reflects inter-annual variability in seasonal transitions, as September separates summer from the beginning of winter. However, methodological differences also play a major role. Methodological influence is particularly evident with respect to the high proportions of MGIIa-L1 in our samples, which has not been observed before in the WSC, and is typical of late summer communities in temperate coastal ecosystems [73]. The previous employment of bacterial-specific 16 S rRNA gene based primers [71] has likely contributed to the MGII, and Archaea more generally, being overlooked in the WSC, wherein they could represent an important fraction of the microbial community.

### Carbohydrate utilisation potential of microbial communities varied across samples

Microbial carbohydrate utilisation potential was assessed through the abundance and transcription of CAZymes. In particular, we focused on those involved in degradative processes (glycoside hydrolase, GH; carbohydrate-binding module, CBM; carbohydrate esterase, CE; polysaccharide lyase, PL).

CAZyme genes represented a minor proportion of community gene content but a higher proportion of gene transcription. The number of CAZyme genes ranged from 7 – 19 per microbial genome, which corresponded to 0.3% of community gene content, on average. In contrast, CAZyme genes represented between 1.5% and 3.0% of community gene transcription. Employing a dissimilarity-based approach, we observed that the CAZyme compositions of samples from S8 and S26 were grouped together based on station (location) whereas those of S10 and S25 showed no coherent clustering (Supplementary Figure S9).

**Fig. 5:**
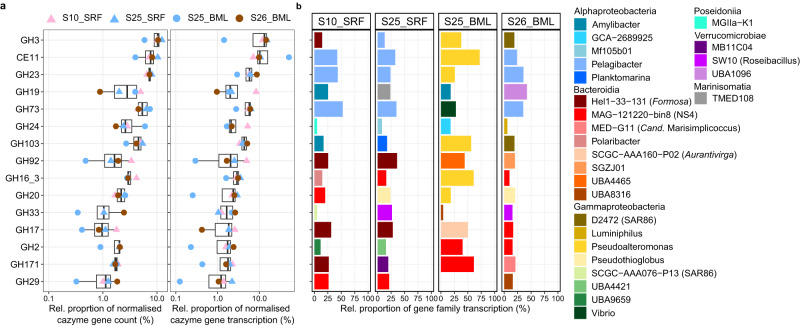
Abundance, transcription and taxonomic information of the CAZyme gene families with the highest proportional transcription across samples. **a** The abundance and transcription of carbohydrate-active enzyme (CAZyme) genes was normalised by the number of microbial genomes per sample and then converted to relative proportions. The selected CAZyme gene families visualised are those that reached the highest proportions of transcription across samples. **b** The genera that contributed the most to transcription of each gene family in each metatranscriptome. Taxonomic classification of CAZyme genes was derived from the classification of the read from which it was derived.

The CAZyme gene profiles comprised a core backbone of universally abundant and transcribed gene families. The most abundant gene families in all samples were those involved in peptidoglycan synthesis and degradation, which is in agreement with previous observations and reflects the core machinery required for bacterial cell membrane construction and maintenance [21]. These core CAZyme gene families included CE11, GH23, GH103 and GH73 that together represented, on average, 27% of CAZyme gene abundance (average of 0.83 PMG) and 35% of CAZyme gene transcription (Fig. 5 and Supplementary Tables S7–S10). Several CAZyme gene families involved in the degradation of algal-derived glycans also represented a high proportion of gene abundance and transcription. Most notable was the GH16_3 gene family, which contains enzymes that degrade laminarin [74], that constituted 3.1% of CAZyme gene abundance and 2.7% of CAZyme gene transcription. Other prominent gene families included those known to target sialic acids (GH33 [75]; ~1.4% of CAZyme abundance and 1.7% of CAZyme transcription), α-mannans (GH92 [76]; ~1.7% CAZyme abundance and ~1.3% CAZyme transcription) and alpha-linked fucose that is common in FCSPs [77] (GH29; ~1.3% CAZyme abundance and 1.2% CAZyme transcription) (Fig. 5 and Supplementary Tables S7–S10). In general, the abundance and transcription of CAZyme gene families showed a positive linear relationship, except in S25_BML (Supplementary Figure S10). However, for some CAZyme families, relative transcription was twofold higher than relative abundance, such as the alginate-targeting PL7_5 [78] in S10_SRF and the galacturonan-targeting GH28 [79] in S26_BML (Supplementary Figure S11).

Although the microbial community analysis was concentrated on the free-living fraction, the target glycans of transcribed CAZyme gene families corresponded to those detected in the POM fraction. For example, the widespread presence of FCSPs and alginate and the transcription of alpha-fucosidases (GH29) and alginate lyases (PL7_5). Glycans that are part of the algae cell belong to the POM pool, but can become part of DOM through cell lysis, viral infection and grazing (a likely process given the presence of copepods in the extracted 18 S rRNA gene data). The same glycan epitopes can thus be present in both POM and DOM, as has been evidenced during phytoplankton blooms [26]. Therefore, the glycans detected in POM here, were likely also available to free-living heterotrophic microbes.

Distinct microbial taxa were responsible for the transcription of CAZyme families in each sample. For CAZyme gene families involved in bacterial glycan recycling, which are universally present in microbes, the transcription was dominated by the most abundant taxa in the community, such as *Pelagibacter* that accounted for ~45% of the peptidoglycan-targeting GH23 family transcription (Fig. 5). In contrast, the taxa contributing the most to transcription of algal glycan-targeting CAZyme families were affiliated with *Bacteroidia*, including *Polaribacter* and NS4 for laminarin (GH16_3), *Formosa* for α-mannan (GH92) and NS4 and UBA8316 for FCSP (GH29). Several of these genera are well known as carbohydrate-degrading specialists in temperate coastal ecosystems and are among the main microbial responders to spring phytoplankton blooms that annually re-occur [10, 21, 80]. The presence and activity of these genera in our samples suggests that they may also be key players in carbohydrate cycling in high-latitude waters and at later seasonal stages.

### Recovery of metagenome-assembled genomes

**Fig. 6:**
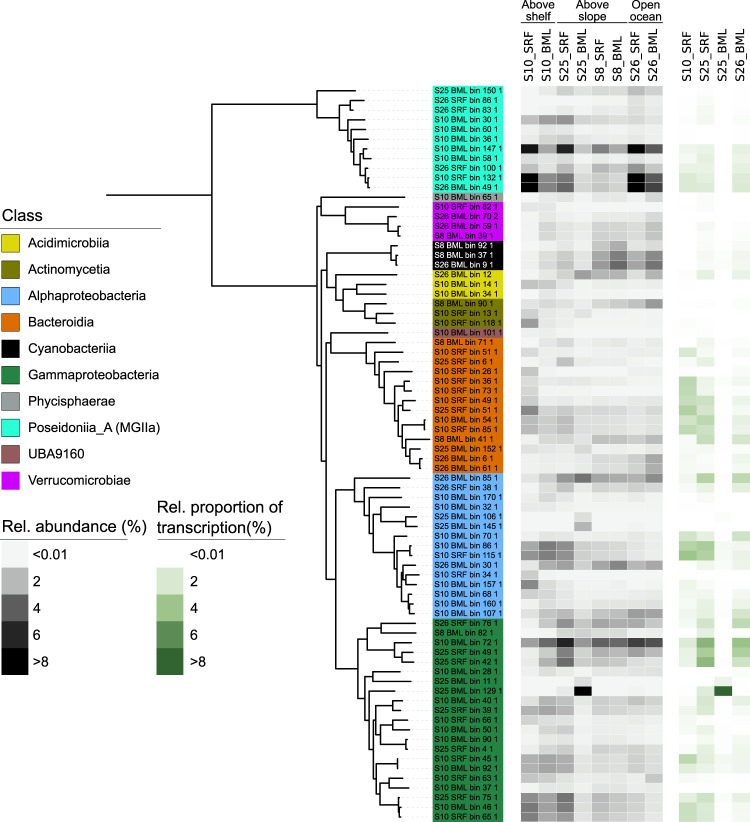
Microbial populations show distinct patterns in distribution and activity across samples. The tree was calculated from a concatenated alignment of 16 single-copy ribosomal protein genes (SC-RBPs), with a threshold of at least 8 genes per MAG for inclusion in the tree. Relative abundance of MAGs was defined as the quotient between the truncated average sequencing depth of the MAG and the average sequencing depth of 16 SC-RBPs in the sample. Relative proportion of transcription was defined by the average TPM value of the MAG-encoded 16 SC-RBPs and the whole sample average TPM values for the 16 SC-RBPs.

To investigate carbohydrate utilisation potential at higher resolution, we next focused on the recovery and analysis of metagenome-assembled genomes (MAGs). A total of 83 population-representative MAGs were recovered, delineated at a 99% ANI threshold (Supplementary Information S1 and Supplementary Table S8). The MAGs captured a substantial fraction of microbial metagenomic reads, 48–88% (Supplementary Table S12), and metatranscriptomic reads, 11–37% (Supplementary Table S13) (Fig. 6). Analogous to the community-level patterns, the coupling of relative abundance and relative proportion of transcription varied across MAGs and taxa. MAGs affiliated with MGIIa-L1 (Marine Group II Archaea) exhibited high relative abundance (up to 12%) but low relative transcription (up to 1.5%) while the *Aurantivirga*-affiliated MAG showed the opposite trend, with ~0.7% relative abundance but ~2.7% relative proportion of transcription. A MAG (S25_BML_bin_129) that shares 97.1% ANI to *Pseudoalteromonas primoryensis* was also recovered. The *Pseudoalteromonas primoryensis* MAG constituted 22.8% relative abundance of sample S25_BML, indicating that the pattern observed at the community-level, and likely resulting from copepod faecal pellet influence, was driven by a single species.

### Carbohydrate degradation gene transcription profiles of microbial populations

**Fig. 7:**
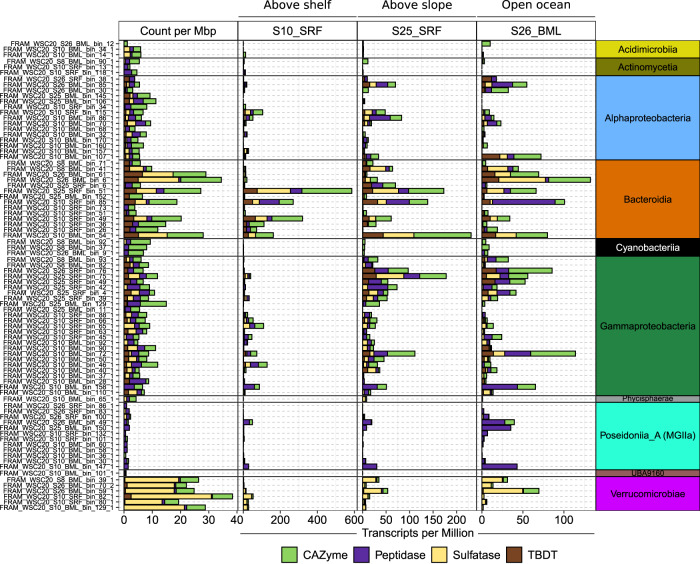
Count and transcription level of carbohydrate utilisation genes for population-representative MAGs. Carbohydrate utilisation genes were clustered into four groups based on function. The count and TPM value of all genes within each group were summed. Gene counts were further normalised by MAG genome size. TBDT = TonB-dependent transporter.

The carbohydrate utilisation potential of populations was assessed through the abundance and transcription of CAZymes in combination with TonB-Dependent Transporters (TBDTs), sulfatases and peptidases. Peptidases were included for comparison as proteins are another key substrate used by heterotrophic microbes. The largest CAZyme gene repertoires were observed in *Verrucomicrobiae*- and *Bacteroidia*-affiliated MAGs, with an average of 15 and 14 CAZymes per Mbp, respectively (Fig. 7). In contrast, *Poseidoniia*-affiliated MAGs harboured few CAZymes, 1 per Mbp, but high peptidase:CAZyme ratios, ~3.7:1. The high peptidase content of *Poseidoniia*- MAGs indicates a preference for proteinaceous substrates, in line with previous observations for this taxon [73]. With respect to sulfatases, *Verrucomicrobiae*-affiliated MAGs harboured the most extensive repertoires, with an average of 24 per Mbp. The observed differences in carbohydrate utilisation potential for these taxa are in accordance with previous findings, with large CAZyme repertoires reported for *Bacteroidia* and a specialisation on sulfated polysaccharides in *Verrucomicrobiae* [38, 77].

In each sample we observed a unique assemblage of populations that dominated carbohydrate-degradation related gene transcription (Fig. 7). In the above-shelf sample S10_SRF, CAZyme, sulfatase and TBDT transcription values were dominated by only a few *Bacteroidia* representatives, particularly *Formosa* (25_SRF_bin_51_1) and NS2b (S10_SRF_bin_49_1). In contrast, the above-slope sample S25_SRF was characterised by a larger number and diversity of populations of the *Bacteroidia* and *Gammaproteobacteria* that exhibited comparable transcription values. The key contributors to CAZyme transcription in S25_SRF included two distinct populations affiliated with the NS4 Marine Group (S10_SRF_bin_85_1 and S10_BML_bin_54_1) along with one of *Formosa* (S25_SRF_bin_51_1), SAR92 (S25_SRF_bin_75_1) and SAR86 (S10_BML_bin_72_1). In addition, high sulfatase transcription in S25_SRF was observed for two populations of *Roseibacillus* (*Verrucomicrobiae*). In sample S26_BML, comparably high CAZyme gene transcription levels were observed across numerous populations assigned to *Bacteroidia, Gammaproteobacteria, Poseidoniia, Alphaproteobacteria* and *Verrucomicrobiae* that were less active in the other samples, which may reflect differences in depth. The largest contributors to CAZyme transcription in S26_BML that were less active in other samples included those assigned to *Cand*. Arcticimaribacter (S26_BML_bin_6_1) [81], *Flavobacteriaceae* (S26_BML_bin_6_1) and *Planktomarina* (S10_BML_bin_107_1). As could be expected, the TPM values for the focal gene groups in S25_BML were strongly dominated by the *Pseudoalteromonas* representative, and thus we did not include this sample in the visualisation. The MAG-based analysis revealed that population’s exhibit sample-specific transcription profiles and suggest that the community-level profiles may be underpinned by only a few populations.

### Disentangling transcription profiles of dominant populations

**Fig. 8:**
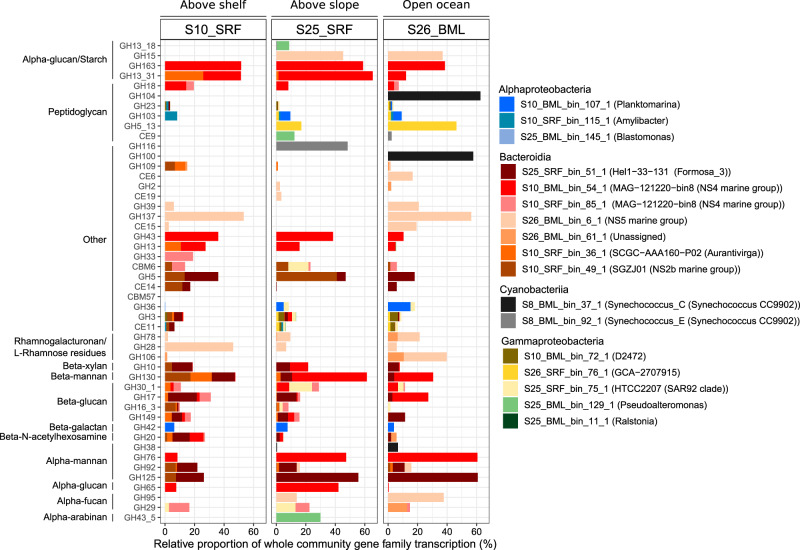
Comparison of CAZyme gene family transcription by selected MAGs across the four metatranscriptome samples. The ten MAGs that exhibited the highest CAZyme transcription values in each sample were selected. Within each of those MAGs, only CAZyme gene families with transcription values higher than ribosomal proteins were retained. To determine the proportion of transcription at the gene family level, we mapped all transcripts identified as CAZymes at the community level to MAG predicted genes. Presented here is the proportion of transcripts of each gene family associated with the selected MAGs – proportion of sample total transcription for that gene family.

To place population transcription into the context of the communities, we determined the proportion of community transcription of each CAZyme gene family by each MAG (see Methods). We further focused on the top six MAGs contributing to transcription of each gene family and within those, only the gene families that were more transcribed than single-copy ribosomal protein genes – considered up-transcribed in relation to the genome (Fig. 8).

Amongst the populations contributing the most to CAZyme gene transcription, we observed gene families that were universally transcribed along with those uniquely transcribed by a single, or few discrete populations. The potential use of communal substrates was evidenced by the similar transcription level of a CAZyme gene family by multiple populations in each sample (Fig. 8). The most notable example was laminarin (GH16_3 and GH17), which is a structurally simple glycan that has previously been shown to be widely accessible to carbohydrate-degrading microbes [10, 17]. In contrast, the community transcription of other CAZyme gene families was dominated by only a few populations that typically varied across samples, including those that target FCSP (GH29 and GH95) and β-xylan (GH10 [82]). For some predicted glycan targets, only a single population was identified as dominating community transcription of the respective CAZyme gene families, such as rhamnogalacturonan (GH28 and GH78) by an NS5 population. The populations showing unique CAZyme gene family transcription patterns are taxonomically related to those that are primary responders to spring phytoplankton blooms in temperate ecosystems and are documented as glycan specialist degraders, such as *Formosa* and the NS4 and NS5 Marine Groups [21]. Therefore, although our limited sampling scope inhibits confident ecological conclusions, it suggests that similar microbial players may be important in glycan degradation at higher latitudes and later seasonal stages, which warrants further investigation.

Microbes that rely on carbohydrates as a main substrate source often have specific genomic arrangements to optimise utilisation, namely polysaccharide utilisation loci (PULs). As such, PULs are often used in targeted studies to assess microbial glycan utilisation and glycan-based niche partitioning in environmental samples [10, 17, 22]. In line with this, we investigated the presence and transcription of PULs within MAGs, but gained little additional insights into potential glycan utilisation by doing so. In populations harbouring multiple PULs, negligible differences in transcription were observed despite their predicted glycan targets being distinct, suggesting little transcriptional regulation. Only in the case of the NS2b representative were distinct PUL transcription levels observed, with a xylan-targeting PUL exhibiting twofold higher transcription than a mannan-targeting PUL in S26_BML (Supplementary Table S14 and S15). The potentially low level of regulation could be indicative of a priming effect, where the detection of one substrate results in transcription of all PULs, however this would need to be investigated over a much larger dataset and complimented by substrate-based incubations.

## Conclusion

In Atlantic waters of the Arctic during late summer, the distribution of POM carbohydrates and their potential utilisation by microbes exhibit variations over spatial scales. The monomeric and glycan composition of POM carbohydrates varied across locations and depths. Typically, higher abundances were observed above the continental slope compared to open-ocean locations. Monosaccharide compositions were dominated by glucose, which decreased in proportion with depth, suggesting preferential utilisation of glucose-based glycans in surface waters. Structurally complex glycans, such as FCSPs that accumulate in POM during phytoplankton blooms, were widely detected, while those with more simple structures, such as laminarin, exhibited patchy distributions. The observed distributions of POM carbohydrates is likely a result of spatial heterogeneity in primary production, as has been described from early summer in this region [47], along with variations in microbial utilisation. Through metatranscriptome analysis, we identified the active fraction of microbial communities and observed variations in carbohydrate degradation-related gene transcription at the community- and population- level across samples. Although gene transcription cannot prove substrate utilisation, we observed that the dominant populations transcribing CAZyme genes belong to the same lineages as microbes that are known to be primary responders to phytoplankton blooms and glycan degraders in temperate ecosystems, suggesting their importance at higher latitudes and later seasonal stages. Furthermore, we observed the up-regulation (transcription higher than that of cellular maintenance machinery) of gene families by specific populations that corresponded to glycans that we could structurally detect in the same sample. In combination, these results provide insights into carbohydrate distribution and potential utilisation patterns by microbes in Atlantic waters of the Arctic during a late summer period. Although our limited sampling scope and inability to replicate samples limits ecological conclusions, the high-quality dataset generated in this study, employing state of the art technologies and protocols, will be of great value in future analysis of marine carbohydrates and the carbon cycle.

### Supplementary information

Supplementary Methods

Supplementary Figures

Supplementary Tables

## Data Availability

The measurements of several abiotic parameters from sensors mounted on the CTD have been published under the PANGAEA accession 943220 [83]. The monosaccharide concentrations have been deposited under the PANGAEA accession 957737. The metagenomic raw reads, assemblies and metagenome-assembled genomes along with the metatranscriptomic raw reads were deposited at ENI-EBA, under the project accession PRJEB58071 (Supplementary Table S16). Tables containing the functional and taxonomic annotation of genes from metagenomic reads and MAGs are provided at 10.17617/3.DZSEAN. Detailed information on how to reproduce our results and generate the figures presented in this manuscript is provided at https://github.com/tpriest0/FRAM_STRAIT_WSC20_data_analysis.
